# Editorial: Role of shoot-derived signals in root responses to environmental changes

**DOI:** 10.3389/fpls.2023.1220592

**Published:** 2023-06-13

**Authors:** María José García, Francisco Javier Romera, Wenna Zhang, Rafael Pérez-Vicente

**Affiliations:** ^1^Department of Agronomy (DAUCO-María de Maeztu Unit of Excellence), Edificio Celestino Mutis (C-4), Universidad de Córdoba, Córdoba, Spain; ^2^Beijing Key Laboratory of Growth and Developmental Regulation for Protected Vegetable Crops, College of Horticulture, China Agricultural University, Beijing, China; ^3^Department of Botany, Ecology and Plant Physiology, Edificio Celestino Mutis (C-4), Universidad de Córdoba, Córdoba, Spain

**Keywords:** long distance signals, MicroRNAs, hormones, phloem peptides, shoot-root communication, environmental changes

Shoot-root communication in plants is essential for the correct integration of responses to environmental changes. As sessile organisms, plants are continuously subjected to changing environmental conditions that in most cases constitute a form of stress. Plants, then must be able to manage the distinct signals perceived by the different organs and produce an integrated response. In this important task, root- and shoot-derived signals play a key role. Shoots and roots deliver messages to each other to induce systemic responses. Shoot growth is modulated by root-derived signals, while nutrient uptake activity in the root is regulated by shoot-derived signals (Notaguchi and Okamoto, 2015; Ko and Helariutta, 2017).

In recent years, several molecules have been identified as systemic signals such as RNAs and microRNAs (Aung et al., 2006; Chiou, 2007; Lin et al., 2008; Buhtz et al., 2010; Liu et al., 2023), small peptides (Koen et al., 2012; Shanmugam et al., 2012; Shanmugam et al., 2015; Grillet et al., 2018; Hirayama et al., 2018; Ota et al., 2020; Kobayashi et al., 2021; Shee et al., 2022; Tabata, 2023), and phytohormones (Kohlen et al., 2011; Borghi et al., 2016; Ko and Helariutta, 2017; Li et al., 2021) that play an essential role in shoot-root communication.

MicroRNAs are 20- to 24-nucleotide RNAs that regulate eukaryotic gene expression posttranscriptionally and transcriptionally as well (by mediating gene silencing) (Brodersen et al., 2008). The knowledge about the implication of shoot-derived RNAs in the regulation of several responses to pathogen and abiotic stresses has been increasing in the last few years and numerous RNAs have been identified. The role of these RNAs in plant development has been thoroughly reviewed very recently by Liu et al. (2023). Thus, we can find shoot-derived RNAs involved in the regulation of root responses to different stresses such as drought [miRNA166 (*O. sativa*) and miRNA390 (*N. tabaccum*)]; chilling [*CmoASCL, CmoSDC, CmoCEK1* (*C. moschata*); *CsaACSL, CsaCEK1, CsaP450s* (*C. sativus*)]; nutritional stresses such as phosphate [miRNA172 (*C. sativus*), miRNA399, miRNA827 and miRNA2111 (*A. thaliana*)] and sulfate [miRNA395 (*A. thaliana*)] deficiencies; and injury response [Prosystemin (PS) (*S. lycopersicum*)] (Liu et al., 2023).

Among the small peptides involved in shoot-root communication are glutathione (GSH; Koen et al., 2012; Shanmugam et al., 2012; Shanmugam et al., 2015) and Iron Man (IMA) peptides (García et al., 2018; Grillet et al., 2018; Hirayama et al., 2018; Gautam et al., 2021; Kobayashi et al., 2021; García et al., 2022; Meng et al., 2022; Peng et al., 2022; Tabata, 2023). Both have been involved in the regulation of the Fe deficiency response. However, while IMA peptides are exclusive for the Fe deficiency response regulatory network, GSH has also been linked to sulfur nutrition (Liu et al., 2009; Koprivova and Kopriva, 2014). Another important peptide in long-distance signaling is the phloem-mobile CEPD-like 2 (CEPDL2) polypeptide which is involved in plant responses to decreased shoot N status. Its expression increases in the leaf vasculature in response to decreased shoot N content and, after translocation to the roots, promotes high-affinity uptake and root-to-shoot transport of nitrate (Ota et al., 2020).

The present Research Topic includes four papers: one review and three original research articles. The review by Bai et al. summarizes the new experimental methods available in the area of synthetic biology to improve the characteristics of natural heavy metal hyperaccumulators and include these characteristics into non-food and high-biomass plant species for phytoremediation of heavy metals. In this review, synthetic biology is presented as an innovative way to build modules with new functions that could be applied to get more efficient natural hyperaccumulator plants in phytoremediation of heavy metals from soil. The authors summarize the new experimental methods for the discovery of synthetic biological elements as well as the knowledge about the construction of circuits and take into account the signaling between the different modules to enable their proper function in the transgenic lines generated.

In Glanz-Idan et al. the importance of a cytokinin (CK) mediated root–shoot communication network is shown in the regulation of leaf senescence. The authors propose that a CK-mediated signal is translocated through the xylem to the leaves where this signal alters CK biosynthesis, resulting in delayed senescence.

García et al. studied the relationship among several regulatory signals related to the Fe deficiency response. IMA peptides were discovered very recently (Grillet et al., 2018; Hirayama et al., 2018) and presented as key regulators of the Fe deficiency response, however their relationship with other known factors that regulate this response, such as ethylene (activating signal) and LODIS (LOng-Distance Iron Signal), a repressive signal (García et al., 2018), was unknown until the publication of this work.

Finally, the third original paper of the present Research Topic by Li et al. attempts to clarify how nitrate inhibits nodule growth and nodule nitrogenase activity in *Glycine max (L.) Merr* roots. The authors employ a dual-root growth system in which both halves of the root are inoculated with rhizobia and only one side is subjected to nitrate treatment. Results obtained suggest that the mechanism by which the plant systemically suppresses nodulation under nitrogen-replete conditions is the reduction of carbon fluxes from shoots to nodules and roots.

To date, most research about plant responses to biotic or abiotic stresses has been carried out in roots or shoots separately without considering possible systemic signals that connect them. However, integration of all signals received from the environment is essential for the correct development of plants, allowing them to respond coordinately and leading to a fine adjustment of their responses in each environmental condition. The knowledge of the signals involved in shoot-root communication will be essential in the near future to developing new plant varieties that are more efficient and better adapted to the changing environmental conditions.

## Author contributions

MG wrote a first draft of the manuscript that was corrected and improved by all the authors. All authors revised the manuscript and approved the submitted version.
